# Classroom Active Breaks to Increase Children’s Physical Activity: A Cross-Sectional Study in the Province of Naples, Italy

**DOI:** 10.3390/ijerph17186599

**Published:** 2020-09-10

**Authors:** Francesca Gallè, Pierluigi Pecoraro, Patrizia Calella, Giuseppe Cerullo, Maria Imoletti, Teresa Mastantuono, Espedita Muscariello, Roberta Ricchiuti, Serena Sensi, Carmelina Sorrentino, Giorgio Liguori, Giuliana Valerio

**Affiliations:** 1 Department of Movement Sciences and Wellbeing, University of Naples “Parthenope”, Via Medina 40, 80133 Naples, Italy; francesca.galle@uniparthenope.it (F.G.); patrizia.calella@uniparthenope.it (P.C.); giuseppe.cerullo@uniparthenope.it (G.C.); roberta.ricchiuti@uniparthenope.it (R.R.); giorgio.liguori@uniparthenope.it (G.L.); 2 Food and Nutrition Hygiene Service, Local Health Authority Napoli 3 Sud, Via Montedoro 47–Torre del Greco, 80059 Naples, Italy; p.pecoraro@aslnapoli3sud.it (P.P.); maria.imoletti@gmail.com (M.I.); teresa.mastantuono@libero.it (T.M.); edy.muscariello@gmail.com (E.M.); serena.sensi@libero.it (S.S.); carmelita24@virgilio.it (C.S.)

**Keywords:** school, physical activity promotion, body mass index, overweight, accelerometer

## Abstract

Background: Classroom Active Breaks (CABs), short active sessions integrated in the school time, have been recognized as a promising tool to reduce sedentary behavior and increase Physical Activity (PA) levels in children. “AulAttiva” is a six-month CABs-based program implemented in primary schools of the province of Naples. The aim of this study was to evaluate its effectiveness by comparing PA and sedentary time of participating pupils respect to a control group, considering also their weight status. Methods: Four third-grade classes, each from 4 schools out of 32 participating in AulAttiva, and 4 third-grade classes, each from 4 schools out of 74 that did not take part, were randomly selected. Finally, 58 children composed the intervention group and 57 the control group. Age, gender, weight and height were registered for each participant. Weight status was classified as non-overweight and overweight/obesity. Sedentary time and PA were assessed through accelerometers along a school day. Results: Light PA was 4 min higher in the AulAttiva group with respect to controls (p = 0.046). Within the non-overweight children, the AulAttiva group spent less time in sedentary behavior and more time in light and total PA than controls. No significant differences were found between the overweight/obese subgroups. Conclusions: The results support the effectiveness of CABs in increasing PA during the school day. Greater effects were registered among normal weight pupils, suggesting the possible influence of weight status on children’s participation to the intervention. Further studies are needed to improve the compliance of overweight/obese children to this intervention.

## 1. Introduction

Children’s levels of physical activity (PA) and time spent in sedentary behaviors are far from the recommendations in Italy, where only 9.5% of boys and 2.6% of girls meet the recommended 60 min/day of moderate-to-vigorous PA [1] and about 50% of 11-year-old children exceed the limit of two hours per day of recreational screen time [2]. Prolonged sedentary time in children may have a significant impact on health, since it is independently associated with weight status and obesity [3]. Indeed, the prevalence of children’s overweight status and obesity in Italy is around 30% [4] and it is among overweight and obesity in Italy is around 30% [4] and it is among the highest in Europe [5]. Therefore, the Italian Ministry of Health released in 2017 an action plan for the promotion of PA for different age groups and settings, underlining the need to reduce sedentary behaviors and prevent excessive weight in children through the collaboration of health, sport and school institutions [6]. Primary schools can play a central role in promoting health behaviors such as PA, since a significant proportion of a child’s day is spent at school, and schools reach all children [7]. However, it has been estimated that European school children spend most time at school in sedentary activities and only a small amount in moderate-to-vigorous intensity physical activities (MVPA), with overweight children less involved in MVPA than normal-weight children [8].

Although physical education may give children an opportunity to practice PA and reduce sedentary time during a typical school day, in several European countries including Italy, the time devoted to this discipline in the primary school curriculum is still flexible and committed to generalist teachers [9]. As such, the time spent in MVPA of a typical physical education session is only 27.3%, while sedentary time represents a similar proportion (24.3%) [10].

A lot of interventions have been implemented successfully in the school setting to reduce sedentary behavior and increase PA levels in children [11]. PA may be offered with different strategies and in different occasions during the school day. Besides the traditional physical education lesson [12], short bursts of MVPA may be proposed within or between periods of academic instruction, through games or exercises involving the whole body behind the desk [7]. In particular, Classroom Active Breaks (CABs) have been recognized as promising tools in this direction [7,13]. CABs are short sessions of PA, usually led by trained teachers, incorporated within the general education classrooms and in the standard classroom time. PA is proposed at any level of intensity, as part of the academic lessons, or through short (5–15 min) PA breaks between lessons or into the main transition periods. They may be implemented in any school context, since they do not require special spaces, equipment or specialized staff.

In a pilot study, we have previously demonstrated the feasibility of the CABs-based intervention “AulAttiva” [14], a program aimed to reduce sedentary time and increase PA in children. This program is included into the action “Schools promoting health” of the 2014–2019 prevention plan of the Campania region, South Italy, and was implemented by the Local Health Authority “Napoli 3 Sud” in some schools of its district within the province of Naples [15]. Since its implementation, the AulAttiva intervention has been progressively introduced in 45 primary schools and involved 6385 pupils attending the third, fourth and fifth grades.

The aim of the present study was to evaluate the effectiveness of the AulAttiva program in increasing children’s PA during the school time by comparing the objectively measured activity levels of a group of pupils involved in the program with those of a matched control group. As secondary aim, we analyzed whether the program generates different responses in terms of PA levels between non-overweight and overweight/obese children.

## 2. Materials and Methods

### 2.1. Participants and Settings

During the 2018–2019 school year, the AulAttiva program was implemented in 32 elementary schools that expressed interest in the territory of the Local Health Authority “Napoli 3 Sud”. It lasted six months, from December 2018 to May 2019. The present cross-sectional investigation was performed in late May at the end of the school year.

**Figure 1:**
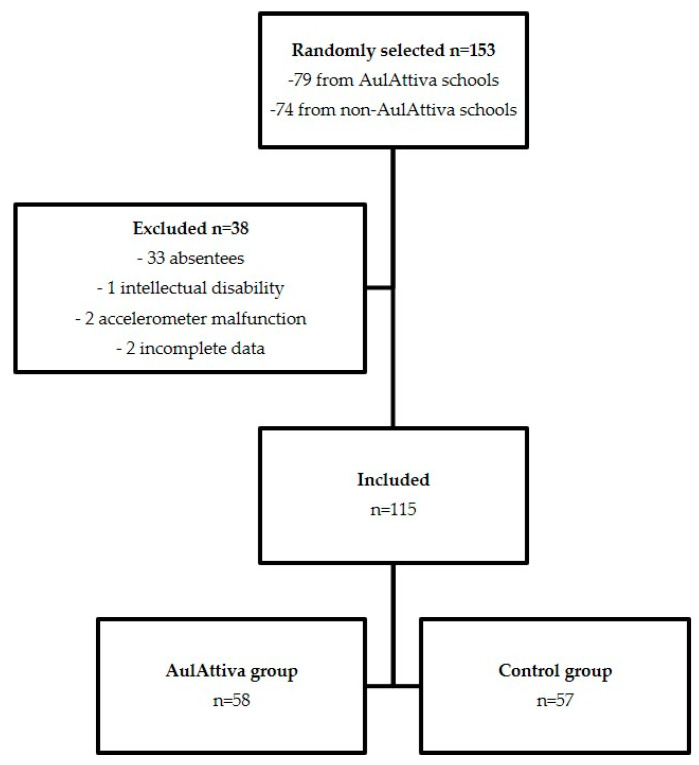
Flow chart of the selection of participants to the study.

Four third-grade classes, each from four different schools out of 32 participating to the AulAttiva program, were selected by simple randomization and composed the intervention group (*n* = 79 children) (here indicated as the “AulAttiva group”). Four third-grade classes, each from four schools out of 74 of the same territory that did not take part to the program, were also randomly selected, and constituted the control group (*n* = 74 children). During a preliminary meeting, a researcher informed parents of these children about the objectives and the methods of the study, and asked them to sign an informed consent. To avoid discrimination, all children attending the selected classes were invited to participate to the study. Subsequently, data from children possibly affected with mobility limitations or diagnosed intellectual disabilities were excluded from the analysis. Complete measures were obtained for 58 children from the AulAttiva schools and 57 pupils from the others. Figure 1 shows an overview of the selection procedure.

The intervention was approved by the Local Health Authority “Napoli 3 Sud” General Management Board (Deliberation n. 820 of 19 October 2018). The study protocol was designed in accordance with the Declaration of Helsinki and approved by the school institution boards.

### 2.2. Procedures

The AulAttiva program was based on the introduction of CABs during school time as previously reported [14]. Briefly, at least one consenting teacher from each class attended a one-day training course to understand the aims of the CABs and the way to perform and supervise the exercise sessions. A researcher demonstrated each exercise in detail, and provided the teachers with both printed materials and videos explaining the proper performance of the suggested tasks. The program consisted into two bouts of CABs in each weekly school day, except the day including physical education or any other outdoor activity. Each bout lasted 5 min and included four exercises focused on fundamental movement skills, light aerobic activity, light strength activity and gross motor coordination. Children performed exercises standing behind their school desk. For the purposes of this study, all the children belonging to the intervention and control groups underwent BMI and accelerometer measurements.

On a planned school day, before the beginning of classroom activities, two examiners who were trained in anthropometric assessment measured children’s height and weight using a Seca 214 portable stadiometer and a Seca 872 medical scale (Seca Deutschland, Hamburg, Germany) under standard conditions, e.g., without shoes and wearing light clothes. Height and weight were measured to the nearest 0.1 cm and 0.01 kg respectively and were used to calculate BMI of each participant. Weight status was classified as non-overweight (BMI < 85th percentile) and overweight/obesity (BMI ≥ 85th percentile of the International Obesity Task Force BMI cut-offs [16].

The day after, children were invited by the same examiners to wear the ActiGraph GT1M accelerometer (ActiGraph LLC, Pensacola, FL, USA) around the waist during the school day (since 8:30 a.m. to 13:30 p.m., ~300 min). Accelerometers were initialized to collect data in 15-s epochs. Activity counts per minute (CPM) were categorized on the basis of the Evenson’s cut points (Sedentary 0–100 counts per minute, Light 101–2295 CPM, Moderate 2296–4011 CPM, Vigorous > 4012 CPM) [17,18]. Minutes spent in sedentary activities and in light, moderate, vigorous and total (sum of light, moderate and vigorous) PA were considered as outcomes.

### 2.3. Data Analysis

The a priori power analysis was carried out considering a reduction of sedentary time of about 12.5 min per school day induced by the intervention, as reported in our previous study [14]. The expected sample size with a confidence interval of 95% and a power of 80% was at least 52 subjects per group: therefore, we considered a total sample size of at least 104 pupils as the minimum required to perform the study.

Data are presented as mean ± standard deviation for parametric variables and as median, 25th and 75th percentiles for non-parametric variables. The Shapiro–Wilk test was used to assess the distribution of the variables. The time spent in sedentary or physical activities was also expressed as percentage of the total school daytime. The chi-squared test was used to compare gender distribution between groups. A Student’s *t*-test was performed to compare data between intervention and control group for parametric variables, while the Mann–Whitney test was used for the others. The significance level was assumed as *p* < 0.05. The Cohen’s *d* value was calculated to assess the effect size for these comparisons (small 0.10–0.40, medium 0.50–0.70, large ≥ 0.80). Analyses were conducted using the IBM SPSS Statistics for Windows, Version 26.0, IBM Corp., Armonk, NY, USA.

## 3. Results

The main characteristics of the two groups and those of the respective BMI subgroups are shown in Table 1. As for the total sample, a significant difference was found between intervention and control groups only for age, but it was non clinically meaningful. The comparison between BMI subgroups showed a higher prevalence of males in control overweight/obese children (Table 1).

Table 2 shows the median and the corresponding 25th and 75th percentile values of time spent by participants in sedentary activities or in light, moderate/vigorous and total PA. In the comparison between the two groups, a significant difference was found only regarding light PA, which was 4 min higher in the AulAttiva group with respect to controls. Within the non-overweight children, the AulAttiva group spent a lower amount of time in sedentary behavior and a higher amount of time in light and total PA than normal weight controls (Table 2). No significant differences were found between the overweight/obese subgroups. Medium effect sizes were registered only for significant comparisons between the normal weight subgroups.

**Figure 2:**
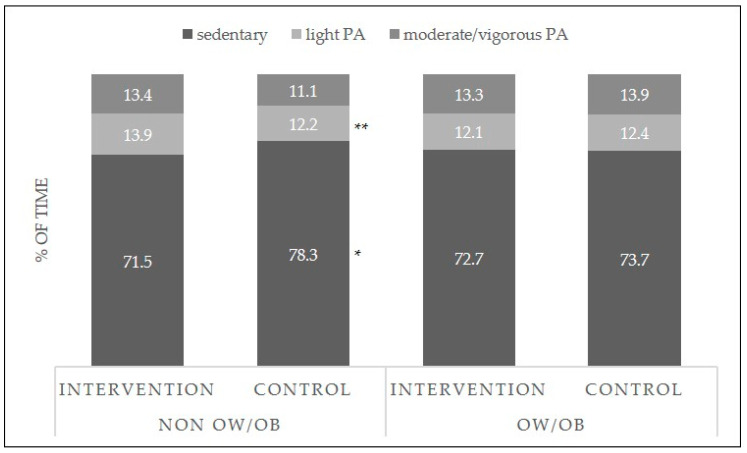
Percent time spent in sedentary or light and moderate/vigorous activities by intervention and control participants grouped by BMI category. ** *p* = 0.012; * *p* = 0.022 versus intervention group (Mann–Whitney test); PA: physical activity; OW: overweight; OB: obese.

Figure 2 shows the percentage of time spent during the school day in sedentary, light and moderate/vigorous activities by AulAttiva and control participants grouped by BMI category. A significantly lower amount of sedentary time and a higher percentage of light PA time were found in non-overweight pupils participating to the program respect to their control counterparts, while no significant differences were found between the two categories within the overweight/obese children.

## 4. Discussion

This study indicated the extent to which a program based on CABs may decrease sedentary time and increase PA levels of children at school. Specifically, the program was able to significantly increase the levels of light PA in the intervention group compared to controls.

The public health potential of school-based PA interventions is highly recognized as a useful strategy to counteract the effects of a sedentary lifestyle [11], which is negatively associated with health indicators [3]. Considering that interrupting sitting with brief moderate-intensity bouts may improve cardiometabolic outcomes [19], CABs may be considered a useful, feasible and acceptable health promotion strategy.

The level of effectiveness of CABs in reducing sedentary and increase physical activities during the school day is still debated, since the available studies show high levels of heterogeneity for samples, intervention characteristics and study design [7,20,21,22]. Through isotemporal substitution modelling that used pooled data of children and adolescents from the International Children’s Accelerometry Database, favorable theoretical associations with most cardiometabolic risk markers in healthy youth were shown when replacing at least 10 min of sedentary time with an equal amount of MVPA, or even light PA, although to a lesser extent [23].

In the present study, we found that children who performed two 5-min CABs in a typical school day, spent about 6 min less time in sedentary behavior and increased their light and MVPA (of about 4 and 2 min) with respect to controls. Our findings show the effectiveness of AulAttiva in increasing time dedicated to PA along the school day in the real world, in agreement with other similar studies [24,25,26,27].

Compared to our previous feasibility study, the expected 12-min reduction in sedentary time found in a pre-post intervention assessment was not achieved. It is possible that the different design of the present study and the adaptation to the study procedures and intervention might have influenced the results, which characterizes the feasibility studies to achieve the most promising outcomes, [28]. It should be noted that, as it occurs in the implementation phase, the program examined in the present study was delivered in a defined context, but one that was not fully controlled. For instance, in the feasibility study a researcher periodically encouraged and supported teachers and school children for a successful implementation of the CABs program and teachers were in touch with the research group at any time for any question or support, while in the implementation phase, it was no longer possible to assure a strict control of teachers’ activity. Similarly, smaller effects on PA (from 19 to 5 min/day) were reported by Carlson et al. [27] when CABs were implemented in a larger sample of schools.

As for BMI analysis, our study showed a significant 5-min decrease in sedentary time, with a similar increase in total PA, in non-overweight children of the intervention group compared to the controls, while no significant differences were found in overweight/obese children.

Studies performed in preschool and elementary school children reported lower PA levels in overweight/obese versus normal weight children [29,30]. Kobel et al. reported that overweight and obese German children spent significantly less time in MVPA than normal weight children during the break times at school [31]. As for CABs, contrasting results were reported. Webster et al. did not find any statistically significant relationship between BMI percentiles and participation in MVPA during CABs [32], while Bershwinger and Brusseau found that obese children had the greatest increase of MVPA compared to normal weight children (6.2 vs. 4.2 min), even if the differences were not significant [33]. Recently, the study by Masini et al. showed longer time spent in MVPA by normal weight respect to overweight/obese Italian children involved in CABs [25].

Excess weight may hinder participation in classroom PA for several reasons, such as lack of self-efficacy, negative body image, and fear of teasing by peers [34,35]. However, most programs are specifically designed for overweight prevention but may also involve children who are already overweight. Therefore, considering the factors that can influence participation in classroom PA interventions is essential to make the programs adaptable to children in any condition.

Our study represents a contribution to the limited body of evidence on the effect of BMI status on children’s participation during CABs. More studies should be designed to understand how to potentially increase the overall PA levels in targeted groups, such as overweight and obese children.

This study has several limitations. First, teachers and children were informed that these would have worn a device to assess their PA levels; therefore, they were not blinded to the experimental outcome. However, it should be underlined that the accelerometer does not provide real-time feedback on PA. Moreover, it has been demonstrated that awareness of wearing an accelerometer had no influence on PA patterns in young people [36]. Furthermore, the size of BMI subgroups and the gender composition of the control overweight/obese subgroup may have influenced the results. Studies involving wider and more representative samples could be useful to confirm our findings. Lastly, we cannot neglect the critical role played by teachers in whether their students chose to participate to the activity breaks. However, as agents of change, the teachers participating to the AulAttiva program were included in the formative stages and were sensitized to the importance of PA for health.

Conversely, the study has some important strengths. First of all, the assessments were done at the end of the school year, when teachers and pupils were familiar with the program. This should be considered because familiarization is essential to maximize movement time and reduce novelty effects. Another strength of this study relies upon the use of accelerometers, which allowed us to objectively measure the time spent in sedentary behavior and in the different levels of PA. This tool showed high ability to compare PA between normal-weight and overweight children without the need of calibration for body weight status [37].

## 5. Conclusions

This study contributes to the characterization of the effectiveness of CABs-based programs as PA promotion interventions in a school setting. The findings support the role of CABs in reducing time spent in sedentary behaviors and increasing PA in children. Greater effects were registered among normal-weight pupils, suggesting the possible influence of BMI on children’s participation to CABs. Further studies are needed to improve the compliance of overweight/obese children to this type of intervention.

## Figures and Tables

**Table 1:** Characteristics of the intervention and control groups in the total sample and by BMI categories.

	Total Sample			Non-Overweight			Overweight/Obese		
	AulAttiva *n* = 58	Control *n* = 57	p	AulAttiva *n* = 36	Control *n* = 30	p	AulAttiva *n* = 22	Control *n* = 27	p
Gender, *n (%)*									
males females	29 (50) 29 (50)	32 (56.1) 25 (43.9)	0.636 ^a^	19 (53) 17 (47)	12 (40) 18 (60)	0.300 ^a^	10 (45.5) 12 (54.6)	20 (74) 7 (26)	0.041 ^a^
Age, (years)	8.8 ± 0.4	8.6 ± 0.3	0.027 ^b^	8.7 ± 0.4	8.6 ± 0.3	0.186 ^b^	8.8 ± 0.3	8.6 ± 0.3	0.057 ^b^
Height, (cm)	133.1 ± 5.9	133.1 ± 5.5	0.982 ^b^	131.6 ± 5.3	131.6 ± 5.6	0.992 ^b^	135.4 ± 6.4	134.7 ± 4.9	0.675 ^b^
Weight, (Kg)	32.9 ± 7.9	34.6 ± 7.9	0.252 ^b^	28.4 ± 3.5	28.7 ± 2.7	0.773 ^b^	40.3 ± 7.4	41.2 ± 6.4	0.629 ^b^
BMI, (Kg/m^2^)	18.4 ± 3.3	19.4 ± 3.7	0.130 ^b^	16.4 ± 1.2	16.5 ± 1.2	0.540 ^b^	21.9 ± 2.8	22.7 ± 2.9	0.315 ^b^

data are expressed as absolute and relative frequencies or means ± standard deviation. BMI: Body mass index; p: *p*-value. ^a^ Chi-squared test; ^b^ Student’s *t*-test.

**Table 2:** Differences in sedentary or physical activity time in the total sample and BMI subgroups with corresponding *p* values and effect sizes.

	Total Sample				Non-Overweight				Overweight/Obese			
	AulAttiva *n* = 58	Control *n* = *57*	p ^a^	d ^b^	AulAttiva *n =* 36	Control *n =* 30	p ^a^	d ^b^	AulAttiva *n =* 22	Control *n =* 27	p ^a^	d ^b^
Sedentary activity (minutes)	187 (174–203)	193 (178–214)	0.126	0.288	186 (173–198)	191 (179–208)	0.022	0.588	189 (174–212)	190 (173–212)	0.896	0.037
Light PA (minutes)	36 (27–42)	32 (23–39)	0.046	0.379	36 (31–44)	35 (27–40)	0.012	0.652	31 (26–42)	32 (24–41)	0.778	0.080
MVPA (minutes)	35 (26–48)	33 (20–46)	0.284	0.201	35 (27–46)	33 (24–45)	0.058	0.479	34 (23–49)	35 (23–48)	0.725	0.101
Total PA (minutes)	73 (57–85)	68 (46–81)	0.129	0.286	74 (62–86)	69 (52–81)	0.022	0.590	71 (48–86)	69 (48–85)	0.856	0.052

Data are expressed as medians (25th–75th percentile). PA: physical activity, MVPA: Moderate/Vigorous Physical Activity; p: *p*-value. **^a^** Mann-Whitney test; **^b^** Cohen’s *d* value.
